# Investigation of Preparation and Shrinkage Characteristics of Multi-Source Solid Waste-Based Cementitious Materials

**DOI:** 10.3390/ma16247522

**Published:** 2023-12-06

**Authors:** Xu Wu, Bo Li, Dingbang Wei, Fucheng Guo, Haidong Ji

**Affiliations:** 1 Gansu Transportation Planning, Survey and Design Institute Co., Ltd., Lanzhou 730030, China; 18893105922@163.com (X.W.); weidingbang@163.com (D.W.); 2 Gansu-Highway Traffic Construction Group Co., Ltd., Lanzhou 730000, China; 3 School of Civil Engineering, Lanzhou Jiaotong University, Lanzhou 730070, China; 13796861108@163.com; 4 Key Laboratory for Special Area Highway Engineering of Ministry of Education, Chang’an University, Xi’an 710064, China

**Keywords:** response surface method, multiple solid waste cementitious materials, compressive strength, synergistic reaction, crack resistance

## Abstract

Cement-stabilized macadam (CEM-SM) base layers on highways are prone to early shrinkage cracking in extremely cold and arid regions, mainly caused by the large drying shrinkage of traditional cement-stabilized base materials. A multi-component solid waste cementitious material (SWCM) was designed based on the response surface method. The synergistic reaction mechanism of SWCM was analyzed using X-ray diffractometer (XRD), Fourier transform infrared spectroscopy (FT-IR) and thermogravimetric analysis (TG). A shrinkage testing system was developed to evaluate the anti-cracking characteristics of stable macadam using multiple solid waste cementitious materials (SWCM-SM), and the strength growth law and frost resistance were analyzed. The results show that the Box–Behnken response surface model was used to obtain the optimal parameter combination for SWCM, including 60% slag, 30% steel slag, and 10% desulfurization gypsum. The compressive strength and flexural strength of SWCM-SM were 24.1% and 26.7% higher than those of CEM-SM after curing 180 days. The frost resistance of SWCM-SM was basically equivalent to that of CEM-SM, and the dry shrinkage strain of SWCM-SM was reduced by 30.7% compared to CEM-SM. It can be concluded that steel slag and desulfurization gypsum stimulate the hydration reaction of slag, thereby improving the bonding strength. Compared to CEM-SM, SWCM-SM exhibits slower hydration reaction and longer hydration duration, exhibiting characteristics of low early strength and high later strength. The early microstrain of the semi-rigid base layer is mainly caused by the occurrence of early water loss shrinkage, and the water loss rate of SWCM-SM is lower than that of CEM-SM. This study concludes that SWCM has good early crack resistance performance for stabilized crushed stones.

## 1. Introduction

The resource utilization of industrial solid waste is receiving increasing attention in the current global warming and energy scarcity environment. Highway engineering is one of the main ways to consume bulk industrial solid waste. Large amounts of industrial solid waste can be used as road aggregates or roadbed fillers to replace the extraction of natural mineral resources. Additionally, solid waste cementitious materials can be developed to replace cement in engineering construction. It can not only reduce the pollution of solid waste storage in the environment, but it can also be an effective way to reduce CO_2_ emissions and promote resource recycling [1].

Solid waste-based cementitious materials are made by mixing various solid waste materials and external activators [2]. The research on solid waste cementitious materials has achieved many breakthrough results after decades of development, and as a green and sustainable material, it provides certain theoretical and technical support for solving global challenges, such as waste treatment, energy scarcity, and ecological environment protection [3,4,5]. Researchers and engineering builders have different opinions on solid waste-based cementitious materials. The overall direction is to use solid waste-based cementitious materials instead of cement for mine filling, heavy metal solidification, instead of concrete for building structures and bridge engineering, and as stabilizers and grouting materials in highway subgrade and pavement.

Kiventera et al. [6] prepared alkali-activated cementitious materials using metakaolin and blast furnace slag as aluminosilicate precursors, which were used to solidify various heavy metal elements (chromium, copper, nickel, zinc, and manganese) in gold tailings to reduce the environmental hazards of tailings storage. Bumanis et al. [7] prepared bulk alkali-activated materials using discarded metakaolin and recycled aluminum and evaluated their potential for inducing precipitation and adsorption to extract heavy metals from wastewater and directly applying them to fill roadbeds. Khalid et al. [8] synthesized geopolymers using fly ash and slag as precursors using a one-step hydrothermal method. The adsorption test shows that the maximum adsorption capacity of the geopolymer for Pb^2+^ is significantly better than the reported large volume adsorbents so far. Uliasz-Bocheńczyk et al. [9] used coal and biomass co-combustion to produce fly ash for mineral sequestration of carbon dioxide. Yu et al. [10] prepared cementitious materials with steel slag, blast furnace slag, and gypsum to solidify heavy metal cadmium. Qing [11] prepared multi-component cementitious materials based on slag, converter slag, refining slag, and gypsum. Two types of concrete materials with different performances have been developed: solid waste-based cementitious material steel fiber-reinforced ultra-high-performance concrete and solid waste-based cementitious material pre-mixed pumped concrete, overcoming the problem of the low early strength of concrete using solid waste cementitious materials. Park et al. [12] studied a high-performance concrete made with specific cementitious materials, where the proportions of slag, fly ash, clinker, and gypsum dihydrate were 72, 10, 10, and 8, respectively. In environments subjected to long-term sulfate attack, the strength and strength growth rate of this cementitious material were higher than 42.5R ordinary Portland cement under the same erosion conditions. Ahirwar et al. [13] studied using fly ash and alkali activators as cementitious materials to replace all aggregates with construction waste to produce recycled aggregate concrete. The results indicate that the 7-day compressive strength and splitting strength of fly ash-based recycled polymer concrete were 6–15% lower than those of ordinary Portland cement concrete. In addition, Menglim et al. [14] used fly ash and slag geopolymer as a stabilizer in highway engineering to stabilize waste asphalt mixture. Anurag et al. [15] used cement solid waste-based cementitious material to stabilize macadam as the base material, and research has shown that adding 30% geopolymer has good crack resistance for the pavement base. Pathak [16] used slag-based polymer-stabilized macadam as the base material and found that the strength of slag-based polymer-stabilized macadam mixed with fly ash met the requirements of road base materials. Compared to cement-stabilized macadam under the same conditions, it had better freeze–thaw resistance and a smaller dry shrinkage coefficient. Arulrajah et al. [17] used fly ash, slag, and calcium carbide residue to prepare geopolymers for stabilizing recycled concrete aggregates and broken bricks. Shen et al. [18] prepared a solidified material using steel slag, fly ash, and phosphogypsum as raw materials, which can be used as a binder for road base materials. Its stable application in road base materials had better water stability than lime soil. Teerawattanasuk and Voottipruex [19] compared and studied the effect of cement and alkali-activated fly ash geopolymer on stabilizing red soil. Through on-site CBR testing, it was found that both met the relevant requirements. Wu [20] used cementitious materials to solidify phosphogypsum to form full solid waste materials for roadbed filling. In summary, solid waste-based cementitious materials can meet performance requirements in the solidification of heavy metals and as cementitious materials for concrete, road base, and roadbed solidification, and they exhibit some superior properties compared to traditional measures, such as the solidification of heavy metal cadmium, the freeze–thaw resistance of road base materials, and sulfate corrosion resistance in concrete. However, there are basically no relevant reports on the research and application of using solid waste-based cementitious materials instead of cement to suppress crack development in highway base layers in extremely cold and arid regions. This study is based on solid waste resources in the western region of China, combined with the early cracking problem of road base materials under extremely cold and arid conditions. We developed a solid waste-based cementitious material to replace cement for stabilizing the base, alleviating the early cracking problem of the base, and extending the service life of roads.

The strength of solid waste-based cementitious materials comes from the synergistic effects among multiple solid waste materials, which depends on the ratio of different solid wastes. Therefore, it is necessary to determine the optimal ratio of solid waste-based cementitious materials through a reasonable design method. Based on the response surface method, Ji et al. [21] prepared a full solid waste cementitious material using blast furnace slag, steel slag, and desulfurization gypsum as raw materials for mine filling. And it has been proven that the response surface method is efficient for optimizing the design of solid waste cementitious materials. Duan et al. [22] used carbide slag, desulfurization gypsum, and steel slag as raw materials, and added NaOH and NaSiO_3_ as alkali activators. Based on the response surface method, a geopolymer cementitious material was prepared to replace cement. Therefore, this study adopted the response surface method to design and study solid waste-based cementitious materials. Overall, there are few previous research reports on using solid waste-based cementitious materials instead of cement to suppress crack development in highway base layers in extremely cold and arid regions, and a corresponding high-precision and low-human-impact shrinkage testing system was lacking.

The purpose of this study was to develop a solid waste cementitious material instead of cement for use as a cementitious material in road base layers in extremely cold and arid areas, to suppress early cracking of the base layer. This study used response surface methodology to design the composition of solid waste-based cementitious materials and analyzed the synergistic reaction mechanism of multiple solid waste cementitious materials using XRD, FT-IR, and TG. The strength growth pattern and frost resistance were analyzed through tests of unconfined compressive strength, flexural tensile strength, and freeze–thaw cycles at different ages. A shrinkage testing system was developed to evaluate the anti-cracking characteristics of stabilized crushed stone using multiple solid waste-based cementitious materials under low-temperature and low-humidity conditions and to simulate the effect of curing humidity on the cracking characteristics of the base layer.

## 2. Materials and Methods

### 2.1. Materials

#### 2.1.1. Chemical Composition of Solid Waste Materials

The multiple solid waste cementitious materials (SWCMs) included blast furnace slag (GGBS), desulfurization gypsum (FGD), and steel slag (SS), all raw materials are sourced from Jiugang Group Co., Ltd. in Jiayuguan City, China. An X-ray fluorescence spectrometer was used to detect the chemical composition of the various raw materials in the solid waste, and the results are shown in Table 1.

As seen in Table 1, the GGBS was mainly composed of CaO, SiO_2_, and Al_2_O_3_, accounting for 35.62%, 36.86%, and 11.12%, respectively. The chemical composition of the FGD was mainly composed of CaO and SO_3_, accounting for 95.22% altogether. The contents of alkaline oxides, such as CaO and Fe_2_O_3_, in the SS was relatively high, accounting for 52.22% altogether.

#### 2.1.2. Phase Composition of Solid Waste Materials

**Figure 1:**
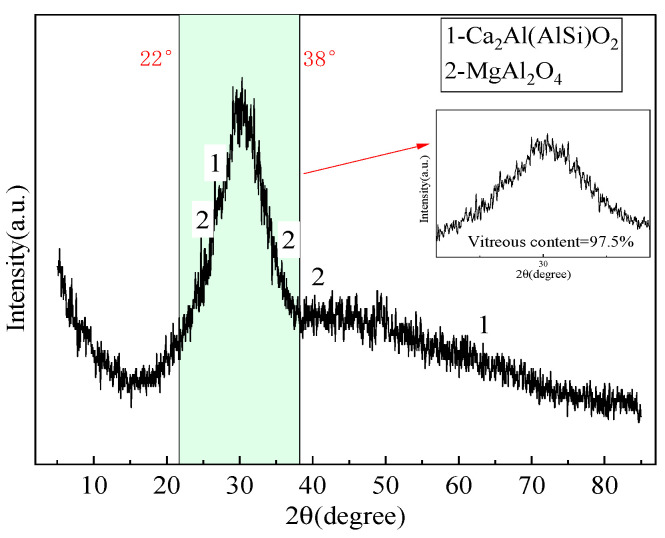
XRD spectrum of GGBS.

**Figure 2:**
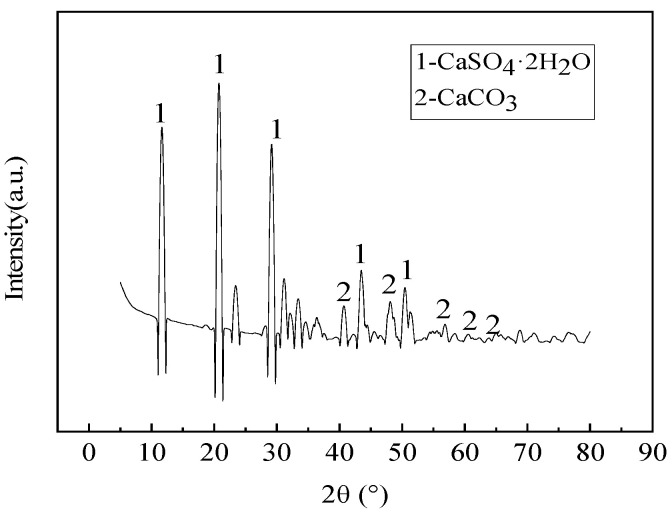
XRD spectrum of FGD.

**Figure 3:**
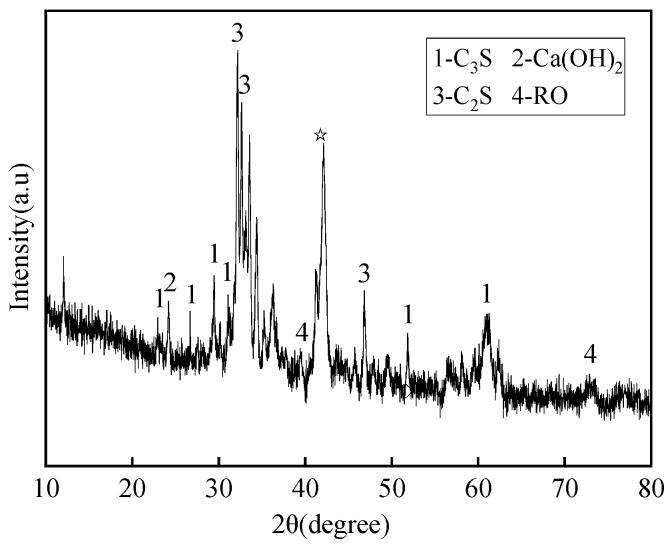
XRD spectrum of SS.

The phase compositions of various industrial solid wastes were tested using X-ray diffraction analysis (XRD). For the GGBS, the testing rate was reduced from 5°/min to 0.8°/min at 22~38° in order to better obtain the amorphous phase content, and the test results are shown in Figure 1, Figure 2 and Figure 3.

As seen in Figure 1, Figure 2 and Figure 3, SS, GGBS, and FGD have the ability to synergistically react [23]. By analyzing the diffraction peaks corresponding to the mineral phases of SS, it was found that its main phase groups were Ca(OH)_2_, tricalcium silicate (C_3_S), dicalcium silicate (C_2_S), etc. C_3_S and C_2_S have the conditions for a hydration reaction, so the SS itself has potential cementitious properties. The silicon and aluminum oxides in the GGBS can react with the Ca(OH)_2_ generated by the hydration of SS, promoting the secondary hydration of the SS and generating more hydrated calcium silicate cement, thereby improving the strength of solid waste mortar specimens. For the GGBS, quantitative analysis was conducted on the content of amorphous glass, which determined the activity of the GGBS. According to *Granulated Blast Furnace Slag Powder for Cement, Mortar, and Concrete* (GB/T18046) [24], the glass phase content in the range of 22 °C to 38 °C was quantitatively analyzed, showing a glass phase content of 97.5% and indicating that the GGBS had high activity. The main mineral phase of the FGD was CaSO_4_·2H_2_O, which had a certain salt excitation effect on the GGBS and SS.

#### 2.1.3. Activity Evaluation of Solid Waste Materials

According to the *Test Method for Strength of Cement Mortar* (GB/T 17671) [25], *Test Method for Activity of Industrial Waste Residue Used in Cement Mixtures* (GB/T 12957) [26], and *Technical Specification for Application of Mineral Admixtures* (GB/T 51003) [27], the activity of various solid wastes was tested, and the results are as follows.

**Figure 4:**
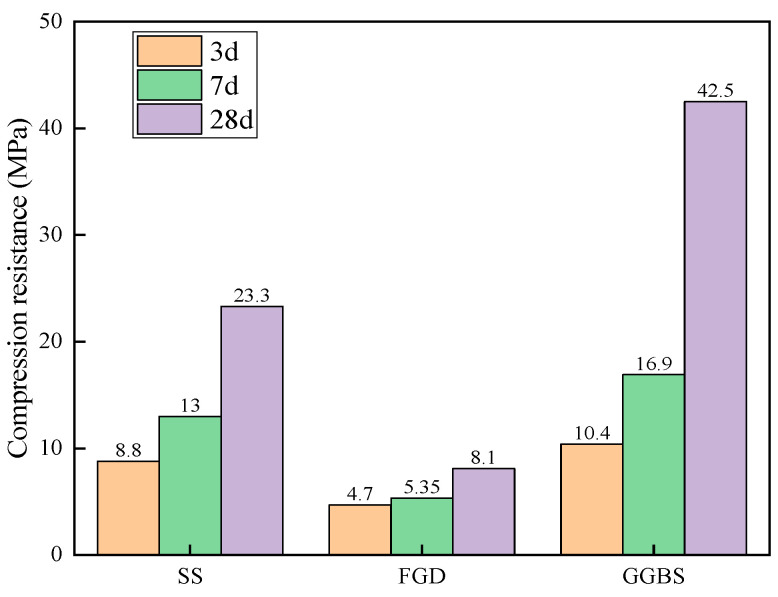
Compressive strength.

**Figure 5:**
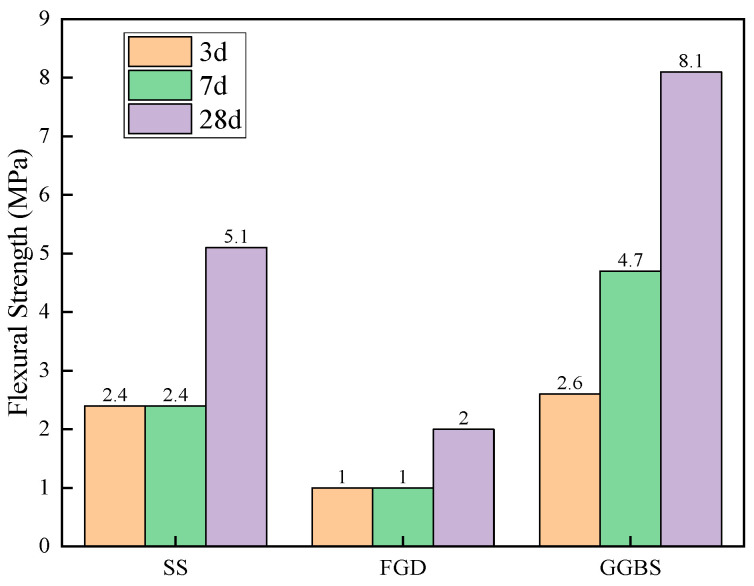
Flexural strength.

As seen in Figure 4 and Figure 5, it can be concluded that the activity order of solid waste ranked as: GGBS > SS > FGD. This can provide a basis for the composition design of solid waste cementitious materials.

### 2.2. Methods

#### 2.2.1. Box–Behnken Response Surface Experimental Design

We selected the dosage of the GGBS, SS, and FGD as independent variables, represented by X_1_, X_2_, and X_3_, and the compressive strength of the SWCMs at 28 days as the response value, represented by Y_1_, Y_2_, and Y_3_. We determined the levels of each factor as follows: GGBS contents of 40%, 50%, and 60%, SS contents of 20%, 30%, and 40%, FGD contents of 5%, 10%, and 15% (all were mass fractions; the same below). The design parameters of the response surface are shown in Table 2.

According to the response surface parameters in Table 2, 15 sets of ratios were designed using Design Expert(13.0.1) software, and 15 ratios of SWCM sand specimens were formed according to standard cementitious sand test methods. The compressive strength was tested after curing under standard conditions for 28 days.

#### 2.2.2. The Synergistic Reaction Mechanism Test of SWCM

(1) XRD analysis

The samples were dried and crushed into powder using a mortar. XRD analysis was performed using an X-ray diffractometer (XRD-7000) from Shimadzu Enterprise Management (Beijing, China) Co., Ltd. The scan range was 5–80°, with a scanning rate of 5°/min. A phase content calculation was conducted using the Rietveld method.

(2) XRF analysis

After drying the sample, a mortar was used to crush it into a powder with a particle size of over 75 microns, and a tablet mold was used to make a test block under high pressure (30 MPa). The XRF analysis was conducted using a sequential X-ray fluorescence spectrometer (XRF-1800) from Shimadzu Enterprise Management (Beijing, China) Co., Ltd. The operating parameters of the instrument were a light tube voltage of 40 kV, a light tube current of 70 mA, and inlet pressure of P10 gas (90% Ar + 10% CH_4_ mixed gas) of 0.75 × 10^6^ Pa, gas flow rate of 1 L/h, and a cooling water control temperature of 17 °C.

(3) FT-IR analysis

After drying the sample, a mortar was used to crush it into a powder with a particle size of over 75 microns, and a tablet press (YY-600) was used to make a test block under high pressure (10 MPa). The infrared spectrometer used in this experiment was provided by Thermo Fisher Scientific (Waltham, MA, USA), and the model was NICOLET-6700. The spectral range was 400~4000 cm^−1^, with 16 scans and a wavenumber accuracy of 0.01 cm^−1^.

(4) TGA analysis

The powder of samples was prepared according to Section 2.2.2 (1). the weight loss of the backfill samples’ powder is tested using a differential thermal and thermogravimetric synchronous analyzer (DTG-60AH) from Shimadzu Enterprise Management (Beijing, China) Co., Ltd. TGA analysis employed a temperature range of 30–900 °C, with a heating rate of 10 °C, a nitrogen atmosphere of 50 mL/min, and a sample mass of around 10 mg.

#### 2.2.3. Mechanical Test of Stabilized Macadam Using SWCM

(1) Mechanical properties test

The procedures for the unconfined compressive strength test were as follows: The specimens were prepared with a density controlled by 98% compaction. The specimen size was a cylinder with a diameter of 150 mm and a height of 150 mm. The specimen was sealed in a bag, and unconfined compressive strength tests were performed under standard curing conditions (temperature 20 ± 2 °C, humidity ≥ 95%) for 7 days, 28 days, 90 days, and 180 days, with a loading rate of 1 mm/min. The test was conducted in accordance with the *Test Specification for Inorganic Binder Stabilized Materials in Highway Engineering* (JTG E51) [28], and the testing equipment was a 3000 KN press (CONCRETO3000X) from Shimadzu Enterprise Management (Beijing, China) Co., Ltd.

The procedure for the bending and tensile strength test was as follows: The bending and tensile strength test piece was a beam-type test piece with a length of 160 mm, a height of 40 mm, and a width of 40 mm. It was cured at a temperature of 20 ± 2 °C and humidity of ≥95%. The bending and tensile strength of specimens aged 28 days, 90 days, and 180 days were tested with a loading rate of 50 mm/min. The test was conducted in accordance with the *Test Specification for Inorganic Binder Stabilized Materials in Highway Engineering* (JTG E51) [28], and the testing equipment was a 3000 KN press (CONCRETO3000x) from Shimadzu Enterprise Management (China) Co., Ltd.

(2) Frost resistance test

The strength loss after freeze–thaw and before freeze–thaw was compared after 10 rounds of freeze–thaw cycle tests, and the frost resistance and durability of stabilized crushed stones cured with different cementitious materials for a certain period of time were evaluated through strength loss. The test conditions were as follows: the freezing temperature was −18 °C, the melting temperature was 20 °C, the freezing time was 12 h, and the melting time was 12 h. The calculation process is shown in Formula (1).

BDR_n_ = R_DC_/R_C_ × 100 (1)

where BDR_n_—compressive strength retention rate (%) of the test piece after n freeze–thaw cycles; R_DC_—compressive strength (%) of the test piece after n freeze–thaw cycles; R_C_—compressive strength of unfrozen thawed specimen (MPA).

(3) Shrinkage test

**Figure 6:**
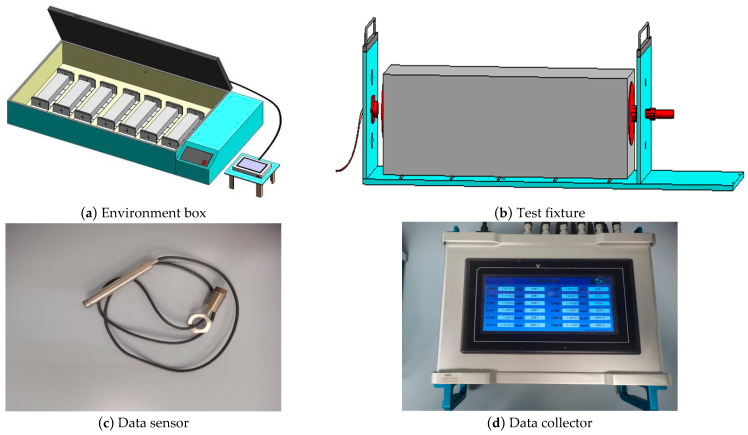
Shrinkage testing system.

**Figure 7:**
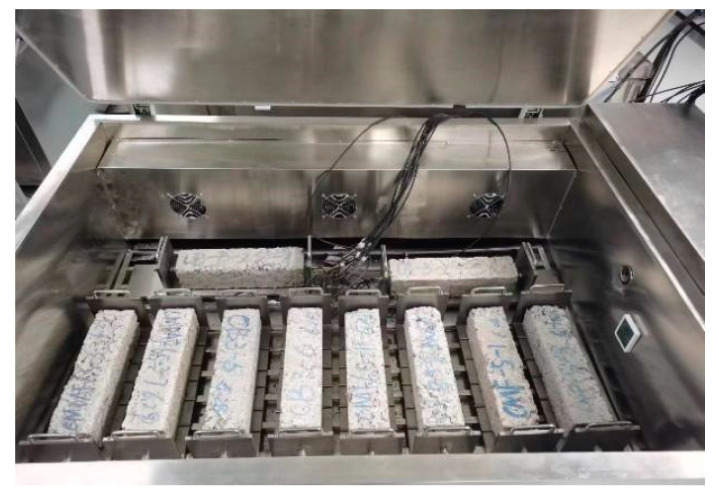
Shrinkage test process.

To evaluate the shrinkage characteristics of SWCM-stabilized macadam (SWCM-SM), a shrinkage test system was developed, as shown in Figure 6. This test system is more convenient and accurate than the traditional test method. This test system consists of four parts: an environment box, test fixture, data sensor, and data collector. The test environment box provides different test temperatures and humidity levels, and the test fixture can test different sizes and types of test pieces. When the test piece is placed on the test fixture, one end of the test piece is fixed to limit its shrinkage, and the other end is connected to a sensor to collect the shrinkage of the test piece. Firstly, the sensor collects the change in the target distance at one end of the specimen and transmits the data to the controller. Then, the controller calculates the shrinkage strain by comparing the change in the target distance with the specimen length. The whole acquisition process had no interference with the test piece and had accurate temperature and humidity control, a long acquisition time, and convenient data acquisition. The testing process is shown in Figure 7.

## 3. Results and Analysis

### 3.1. Analysis of Response Surface Method Results

We used Design Expert(3.0.1) software to perform multiple regression fitting on the test results and established a 28 days compressive strength response surface function equation, as shown in Formula (2). The linear correlation fitting of the model was performed using this equation, and the fitting results are shown in Table 3.

Y_28_ = 28.7 + 2.21X_1_ + 2.17X_2_ − 1.11X_3_ + 0.575X_1_X_2_ − 0.45X_1_X_3_ + 0.475X_2_X_3_ − 0.3X_1_^2^ − 0.92X_2_^2^ − 1.1X_3_^2^ (2)

**Figure 8:**
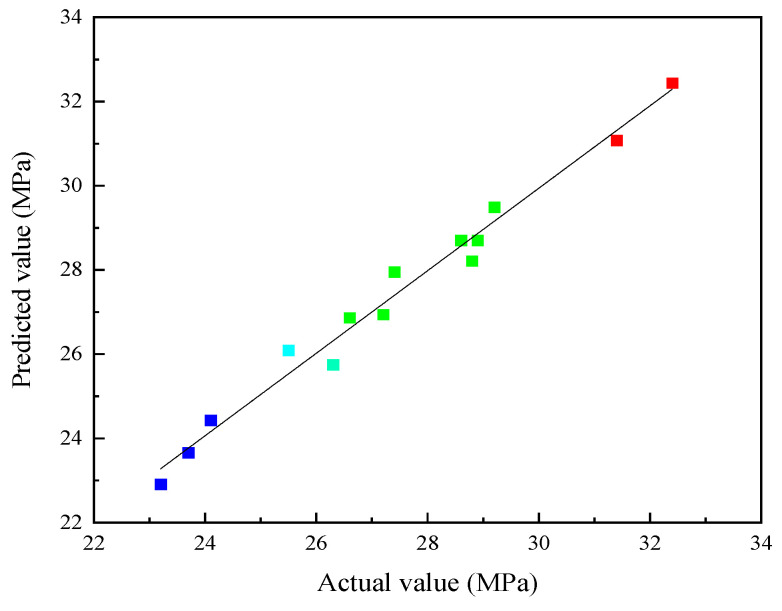
Comparison of experimental values and predicted values of the response surface model.

As seen in Table 3**,** the complex correlation coefficient was 0.9811, the corrected correlation coefficient was 0.9734, and the predicted correlation coefficient was 0.9136. The values shown in Figure 8 confirm the high accuracy and strong reliability of the model.

To optimize the optimal ratio of each component in the multi-component solid waste cementitious material, the 28 d compressive mathematical model was solved using the data optimization function of Design Expert(3.0.1) software. The optimal parameter combination for the mix ratio was 60% GGBS, 30% SS, and 10% FGD. To verify the accuracy and reliability of the response surface model calculation results, five sets of parallel experiments were conducted, with experimental results of 27.5 MPa, 28.4 MPa, 29.1 MPa, 27.9 MPa, and 28.4 MPa, with a relative error of 1.56% (smaller than 5%), which is basically consistent with the predicted values of the model. The reliability of the regression model was verified from both theoretical and practical perspectives.

**Figure 9:**
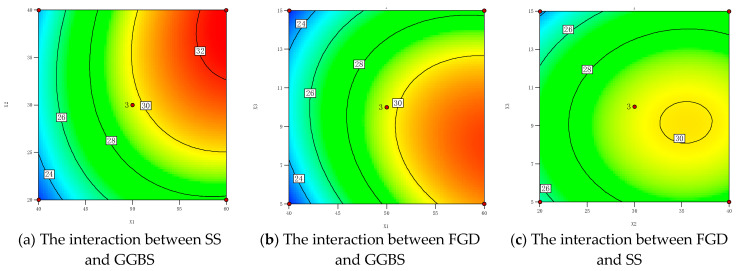
Influence analysis of strength response surface factor interaction after 28 d.

To more intuitively illustrate the correlation between the contents of SS, GGBS, and FGD and the compressive strength of cementitious materials, according to the regression model, we drew a response surface graph of compressive strength with changes in the factor levels, as shown in Figure 9. The analysis results show that the significance order of the influence of independent variables on the 28 days compressive strength was X_1_, X_2_, and X_3_. The order of significance for the interaction of factors was X_1_X_2_, X_2_X_3_, and X_1_X_3_. Figure 9a shows the interaction between the SS powder and the GGBS powder when the FGD content was 10%. The analysis shows that the GGBS had a greater impact on strength compared to SS, but there was a synergistic reaction mechanism between the two, and neither was indispensable. When the content of GGBS was low, the compressive strength showed a slight increase with the increase in the SS content. On the one hand, this indicates that the effect of the SS content on strength was not significant; on the other hand, with a small amount of SS added, the pH value in the hydration system was low, and the GGBS activity was not fully stimulated, resulting in lower strength and a slow increase in strength. With the increase in the SS content, the alkalinity of the hydration system increased, and higher concentrations of OH^−^ rapidly diffused. This accelerated the dissolution and dispersion of the GGBS, induced the fracture of silicon and aluminum oxygen bonds in the GGBS glass body, improved the dissolution rate of cementitious active ions, and promoted the continuous hydration of cementitious materials into hydration products, providing a source of strength for cementitious materials. Figure 9b shows the effect of the interaction between the GGBS and FGD content on the 28-day compressive strength when the SS content was 30%. The analysis shows that the compressive strength significantly increased with the increase in the GGBS content. The FGD rapidly dissolved in the early stage of hydration, and the generated SO_4_^2−^ reacted with the Ca^2+^ and Al^3+^ in the GGBS to promote more AFt generation, which significantly improved the early strength. At the same time, gypsum can promote the hydration of tricalcium silicate (C_3_S) to produce hydrated silicic acid (C-S-H) gel with a stable structure. The interweaving of AFt and C-S-H gel to form a relatively dense structure helped to improve the compressive strength. Figure 9c shows the effect of the interaction between the SS and gypsum content on the 28 day compressive strength. The analysis shows that there was no high strong response zone, indicating that the interaction between SS and gypsum was not significant. Gypsum played an acidic role in the cementitious material, while SS played an alkaline role. Both activators accelerated the alkaline activation reaction of GGBS [29].

### 3.2. Analysis on the Strength Mechanism of SWCM

#### 3.2.1. XRD Analysis of SWCM

**Figure 10:**
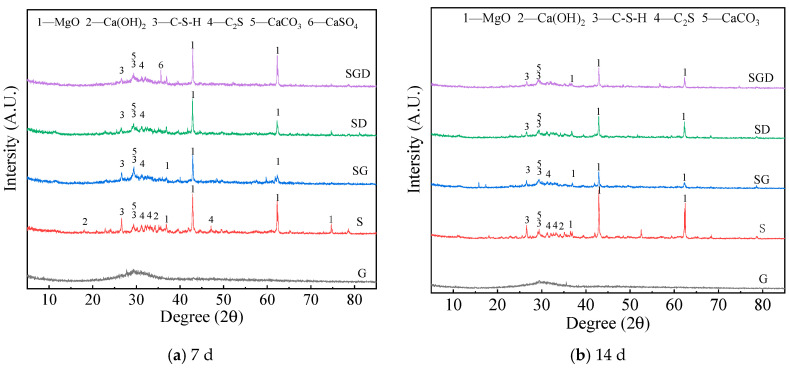
XRD patterns of SWCM at different ages.

To investigate the synergistic effect of solid waste on the mineral composition of cementitious materials at different ages, X-ray diffraction analysis (XRD) was used to test the mineral compositions of cementitious materials, and the results are shown in Figure 10.

As seen in Figure 10, the chemical activity of the SS, mineral composition, and FGD was low, and they did not have hydraulic properties or only had potential hydraulic properties. In the early stage of the hydration reaction, only a small portion of active components participated in the hydration reaction to generate a small amount of hydrated calcium silicate crystals and other amorphous hydration products. This resulted in the lower early strength of specimens with different mix ratios, and there was no significant change in their phase composition during the 7 days and 14 days of curing.

In the SG, SD, and SGD systems, the active components involved in the hydration reaction come from the amorphous calcium silicate aluminate active components in the GGBS [30]. The SS had a higher calcium content compared to the GGBS, but the content of active components in SS was lower. It can be seen from the XRD diffraction pattern of the SD that the addition of SS can introduce more active calcium components into the reaction system, which promotes the early hydration reaction of the system and generates more Ca (OH)_2_ and C-S-H gels [31]. In addition, the SGD specimens exhibited the highest strength after curing for 7 and 14 days compared to the SG specimen. The addition of FGD promoted the synergistic hydration reaction of the SS and mineral powder in the SD system. From the perspective of participating in the reaction, the FGD itself participated in the reaction of the cementitious system, generating ettringite crystals. In addition, from the perspective of activity excitation, the addition of FGD provided SO_4_^2−^, which can also change the S/C of C-S-H and promote the conversion of C-S-H with a high silica/calcium ratio to gel with a low silica/calcium ratio [32,33].

#### 3.2.2. FT-IR Analysis of SWCM

**Figure 11:**
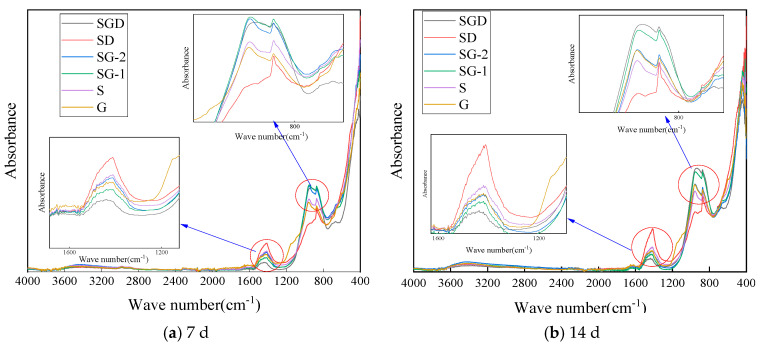
FT-IR spectra of SWCM at different ages.

Infrared absorption spectroscopy is a method for identifying the molecular structure of substances using the characteristic absorption of infrared radiation by molecules. FT-IR spectroscopy can be used to characterize the structural changes in the different silicoaluminate raw materials and hydration products. The test results are shown in Figure 11.

It can be seen in Figure 11 that there was no significant difference in the infrared absorption spectra of the cementitious material specimens with different proportions after curing for 7 days and 14 days This is because the activity of raw materials, such as SS and GGBS, is low, and there was no significant difference in the type and quantity of hydration products after 7 and 14 days of curing, which also makes the early compressive strength of the cementitious materials with different proportions basically the same. The early hydration products of the S, G, SG and SGD systems are also mainly C-A-H and C-S-H, which produced a significant Si-O stretching vibration peak at 969 cm^−1^. According to the infrared absorption peak of SGD, the FGD promoted the formation of ettringite, and the stretching vibration peak of Si-O in ettringite appeared at 1150~1200 cm^−1^ [34].

#### 3.2.3. Thermogravimetric TG Analysis of SWCM

**Figure 12:**
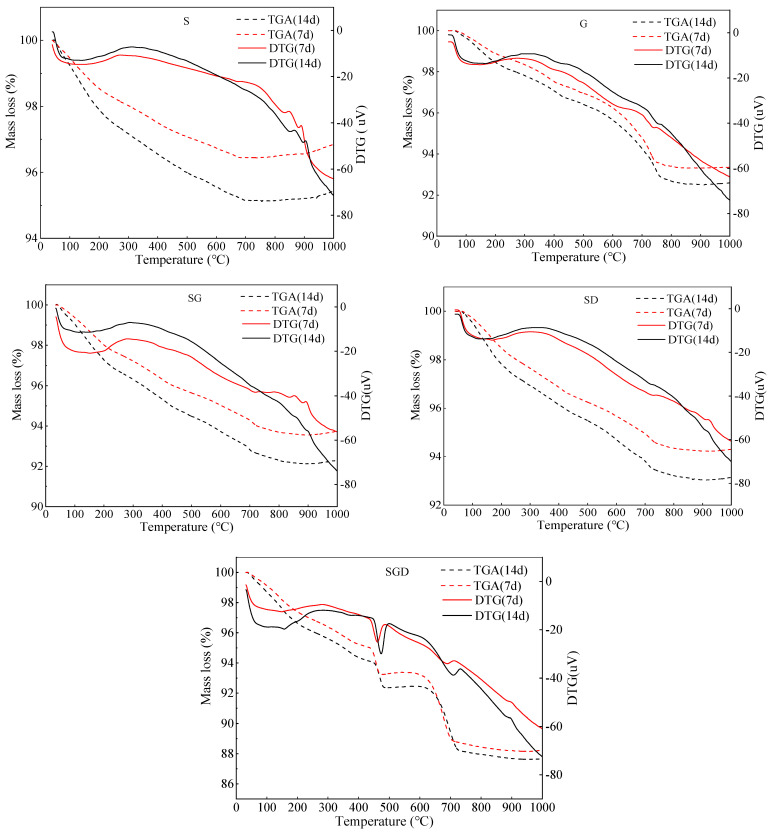
TG spectra of SWCMs at different ages.

An TG spectrometer was used to test and analyze the structural changes of the hydration products in the SWCMs, and the results are shown in Figure 12.

As seen in Figure 12, the TG analysis curve shows that the decomposition weight loss peak of Ca(OH)_2_ was between 380 °C and 450 °C. At the temperature of 450–600 °C, the weight of the specimen was slightly increased due to the transformation of the crystal phase in the SGD system, and the FGD introduced more active calcium components into the reaction system, which promoted the early hydration reaction of the system and generated more hydration products, such as C-S-H. The mass loss of the SGD system increased significantly compared to the G, S, SG and SD systems. The weight loss and content of the C-S-H in the SGD system are the most, indicating that the degree of reaction is higher, and the hydration is more thorough. Compared to other solid waste cementitious material systems, the synergy effect between the solid waste materials in the SGD system is better [35,36].

### 3.3. Analysis of Mechanical Properties of SWCM-SM

The mechanical property evaluation of the SWCM-SM included the strength growth law and strength loss under freeze-thaw cycles. Traditional cement-stabilized macadam (CEM-SM) was used as a blank control group for comparative analysis.

#### 3.3.1. Analysis of Strength Growth Law

**Figure 13:**
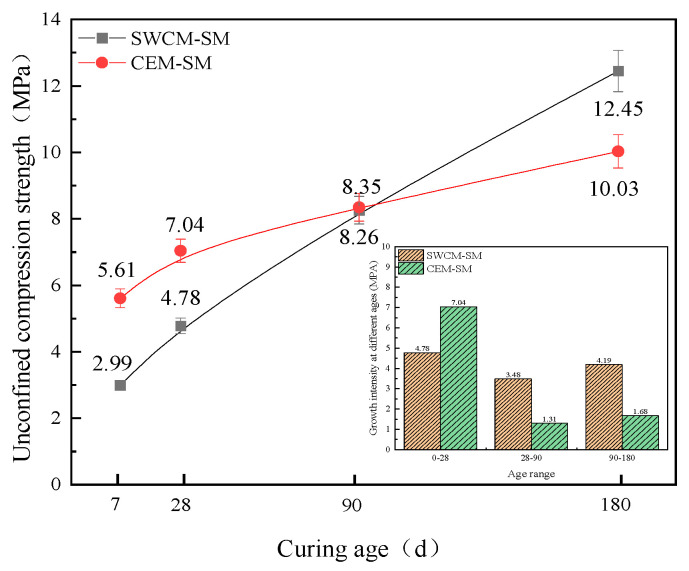
Variation of compressive strength with curing age.

Figure 13 shows that the compressive strength of CEM-SM was higher than that of the SWCM-SM at 7 and 28 days, but the growth trend of the CEM-SM strength became slow after 28 days, while the strength of the SWCM-SM increased linearly. The unconfined strength of the SWCM-SM was basically the same as that of the CEM-SM when cured to 90 days. The unconfined compressive strength of the SWCM-SM was 24.1% higher than that of CEM-SM when cured to 180 days.

**Figure 14:**
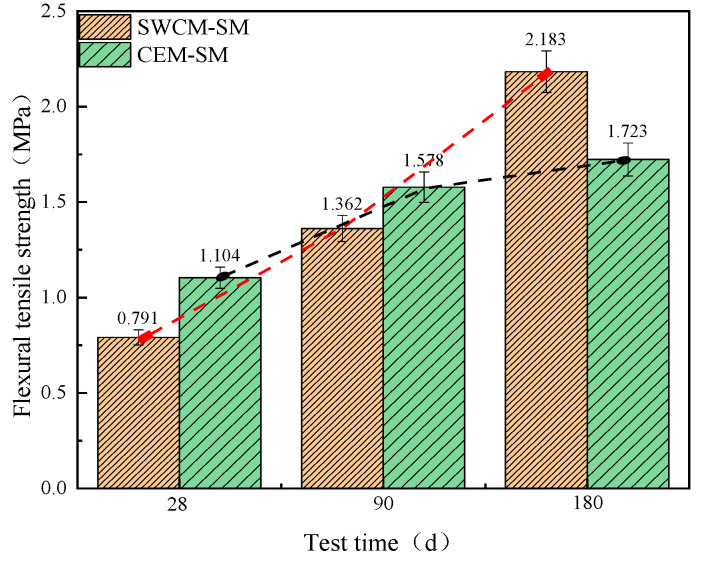
Variation in flexural tensile strength with curing age.

Figure 14 shows that the flexural tensile strength of the CEM-SM was higher than that of SWCM-SM when cured for 28 days and 90 days. The flexural tensile strength of the SWCM-SM was 26.7% higher than that of the CEM-SM when cured for 180 days. The growth trend of flexural tensile strength with age was basically similar to that of the compressive strength, showing that the CEM-SM was high in the early stage and slow in the late stage, and the SWCM-SM was slow in the early stage and high in the late stage.

Therefore, the strength growth trend of the CEM-SM was rapid at first and then slow, while the strength of the SWCM-SM continued to show linear growth. The strength of the SWCM-SM was higher than that of the CEM-SM in the later stage.

The reason for the strength increase was analyzed. The results show that the cement was 42.5 grade ordinary Portland cement, with a strong alkaline environment and fast hydration reaction. The hydration reaction basically reached more than 80% at 28 days. However, the alkaline environment of the SWCMs was weak, which required some cement hydration reaction and gypsum reaction to form excitation conditions, thereby stimulating the formation strength of the GGBS. The hydration reaction process lasted for a long time, resulting in a long duration of strength growth.

#### 3.3.2. Frost Resistance Durability Analysis

**Figure 15:**
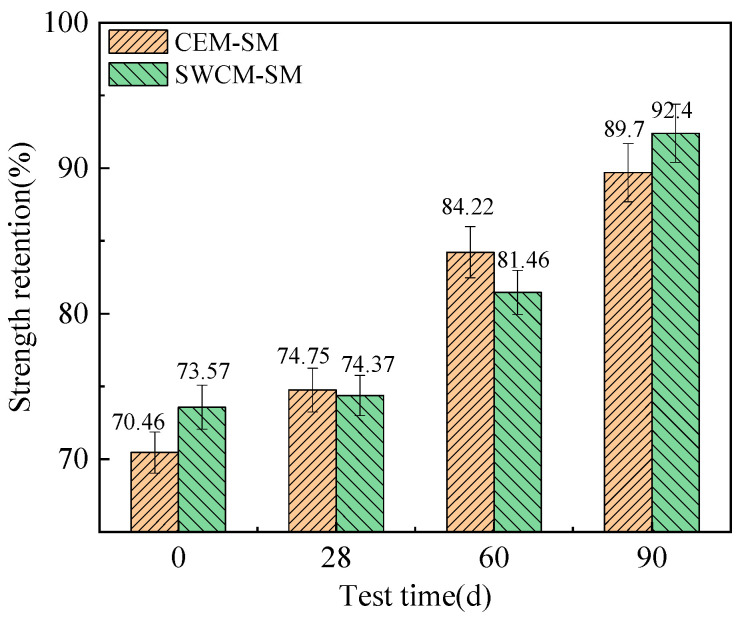
Comparison of freeze–thaw strength retention rate.

In this study, the freeze–thaw strength retention rates of two kinds of cementitious material-stabilized macadam after curing for 28 d, 60 d, 90 d, and 180 d were tested, and the differences in frost resistance were compared and analyzed, as shown in Figure 15.

As seen in Figure 15, the strength loss and residual strength of two kinds of cementitious material-stabilized macadam after 10 freeze–thaw cycles ere compared. The strength retention rate increased with the curing age, which was related to the improvement of its own strength. The strength retention rate of the two kinds of cementitious material-stabilized macadam had little difference at different ages, and the biggest difference was that the SWCM-SM was 3.4% higher than the CEM-SM at 60 days of curing. Therefore, it is considered that the frost resistance of the CEM-SM was basically equivalent to that of the SWCM-SM.

#### 3.3.3. Shrinkage Characteristic Analysis

**Figure 16:**
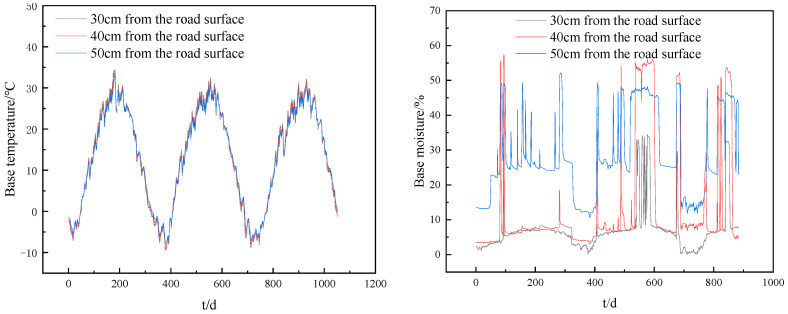
Variation laws of temperature and humidity of pavement base layer year by year.

**Figure 17:**
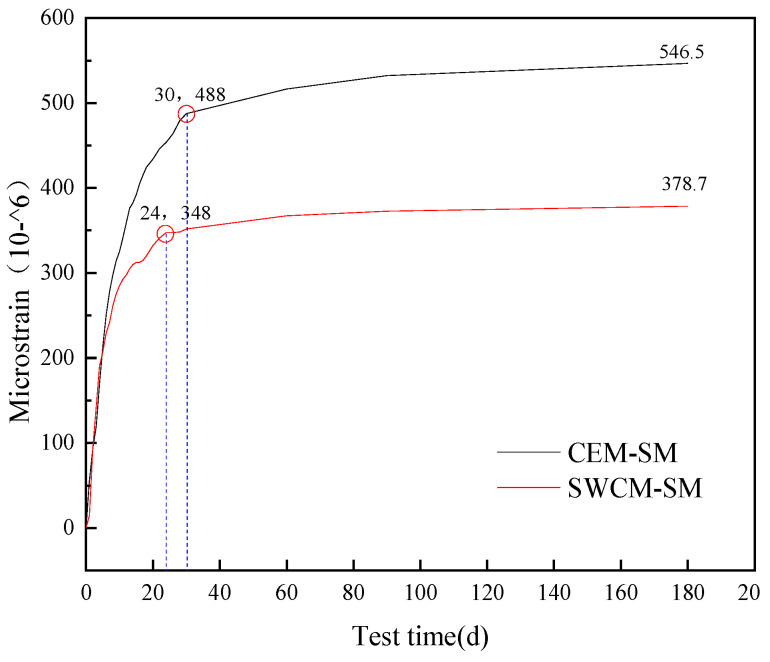
Dry shrinkage strain test results.

The Hexi region of Gansu Province is located in arid and semi-arid climate regions with large temperature differences. The temperature and humidity data of the base were obtained through meteorological observation, as shown in Figure 16. The maximum temperature of the base course is 35 °C, and the humidity of the base course varies greatly, with the maximum humidity of 58% and the minimum humidity of 0. To more accurately evaluate the anti-cracking effect of a SWCM-SM base in an extremely harsh environment, the base course temperature is 35 °C and the humidity is 30%. We tested for excessive shrinkage changes at this temperature and humidity, and the test results are shown in Figure 17.

As seen in Figure 17, the dry shrinkage strain in 180 days was tested, and the dry shrinkage microstrain of two kinds of cementitious material-stabilized macadam was compared. It was concluded that CEM-SM > SWCM-SM, and the dry shrinkage strain of the SWCM-SM was 30.7% lower than that of the CEM-SM. At the same time, it was concluded that the turning point of the growth trend of the dry shrinkage strain of the CEM-SM was the curing age of 30 days, and that of the SWCM-SM was 24 days, so the curing age can be guided according to the turning point of the dry shrinkage strain.

To verify that early microstrain is mainly caused by drying shrinkage, the CEM-SM and SWCM-SM, after curing for a certain period, were respectively watered, and microstrain changes after watering were observed, as shown in Figure 16 and Figure 17.

**Figure 18:**
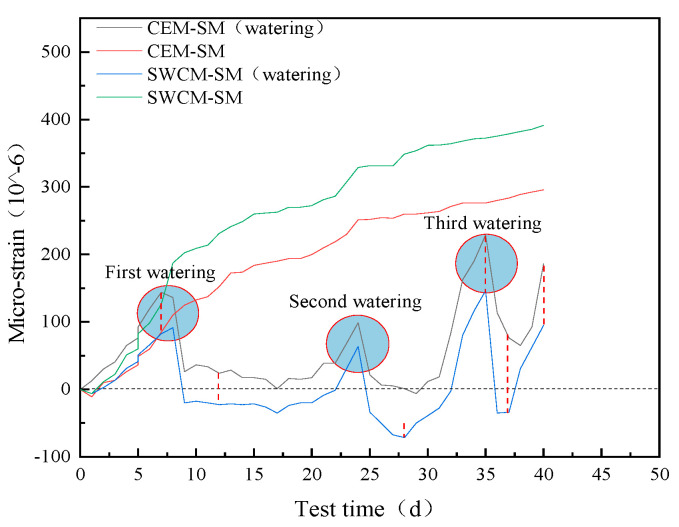
Effect of watering on shrinkage microstrain.

As seen in Figure 18, the two kinds of cementitious material-stabilized macadam specimens were watered for 7 days, 24 days, and 35 days to make them all wet. The microstrain decreased dramatically and returned to the initial state, or micro-expansion occurred after watering, and the same rule applied no matter how old the specimen was. However, the specimen without watering had drying shrinkage, which is enough to prove that the early microstrain was mainly caused by the early water loss shrinkage of the specimen. In addition, the microstrain of the CEM-SM was greater than that of the SWCM-SM whether it was watered or not. This part of the microstrain difference was mainly caused by the different self-shrinkage produced by the hydration reaction between the CEM and SWCM, which shows that the self-shrinkage of the SWCM was less than that of the CEM. Another discovery was that the microstrain of the SWCM-SM was negative after watering, indicating that the specimen had micro-expansion that corresponded to the micro-expansion characteristics of the SWCM itself and further reduced the shrinkage strain.

**Figure 19:**
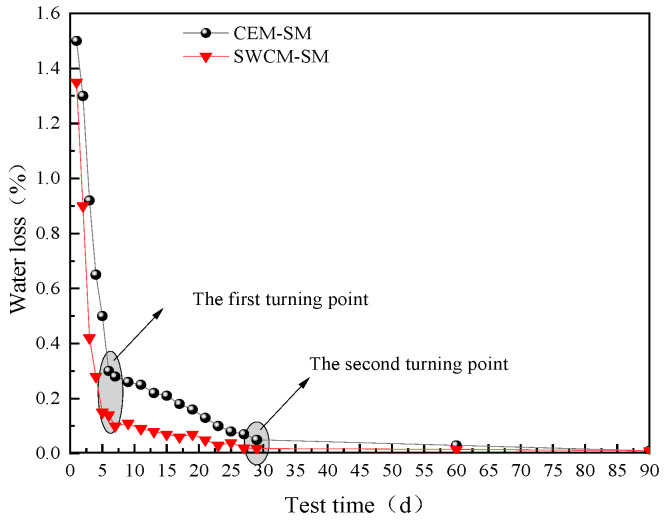
Variation process of water loss rate with time.

Figure 19 shows that the water loss rate of the SWCM-SM was lower than that of the CEM-SM, because the SWCM contained an internal curing agent, which had the effect of absorbing water and moisturizing, so it reduced the large drying shrinkage strain caused by rapid water loss. In addition, water loss is basically divided into three stages: 0–5 days as the first stage, 5–30 days as the second stage, and the third stage is after 30 days. The first stage has the fastest water loss, followed by the second stage, and the third stage has basically no water loss. Water loss mainly occurred after 30 days. Therefore, the main reason for the early shrinkage cracking of the base was caused by water loss shrinkage.

## 4. Conclusions

This article determined the optimal proportion of SWCMs composed of slag, steel slag, and desulfurization gypsum based on the response surface method, deeply explored the influence of the strength formation of SWCMs, and analyzed the mechanical properties of stable base layers of SWCMs and their anti-cracking effects under low-humidity and low-temperature conditions. Some insights and conclusions are as follows:

(1) A response surface function model was established between 28 days compressive strength and multivariate solid waste, with a correlation of 0.9136. The optimal parameter combination of the SWCMs was calculated using this model, which included 60% slag content, 30% steel slag content, and 10% desulfurization gypsum content.
(2) The active ingredients involved in the hydration reaction came from the amorphous calcium silicate aluminate active ingredients in the GGBS. Although SS had a higher calcium content, the active ingredient content in SS was lower. The active calcium component of SS promoted the early hydration reaction of the GGBS, and the FGD participated in the reaction of the cementitious system to generate ettringite crystals, improving the early strength.
(3) The unconfined compressive strength and flexural tensile strength of the SWCM-SM exhibited characteristics of low early strength and high later strength. The frost resistance of the SWCM-SM was basically equivalent to that of the CEM-SM in the entire maintenance cycle.
(4) The dry shrinkage strain of the SWCM-SM was reduced by 30.7% compared to the CEM-SM after 180 days of testing. The turning point for the growth of the dry shrinkage strain of the CEM-SM was 30 days, while that of the SWCM-SM was 24 days. The curing age during the construction process can be determined based on the turning point of the shrinkage strain.
(5) Early microstrain was mainly caused by the early shrinkage of the specimen due to water loss, and the water loss rate of the SWCM-SM was lower than that of the CEM-SM. A portion of the microstrain was contributed by the self-shrinkage generated by the hydration reaction of cementitious materials, and the self-shrinkage of the SWCM was smaller than that of cement.

## Figures and Tables

**Table 1:** Chemical composition of raw materials.

Material	Mass Fraction/%							
	CaO	Al_2_O_3_	SiO_2_	Na_2_O	MgO	Fe_2_O_3_	SO_3_	Other
GGBS	35.62	11.12	36.85	0.56	9.05	0.47	0.11	6.22
SS	40.03	6.16	25.53	0.16	9.03	12.19	3.58	3.32
FGD	41.96	0.71	1.55	0.38	0.22	0.35	53.30	1.53

**Table 2:** Code and level of response surface design factors.

Code Value	Code Level		
	−1	0	1
X1	40	50	60
X2	20	30	40
X3	5	10	15

**Table 3:** Model correlation evaluation.

Evaluation Index	Correlation Evaluation
Complex correlation coefficient ($R^{2}$)	0.9811
Correction correlation coefficient ($R^{2}{}_{Adi}$)	0.9670
Predictive correlation coefficient ($R^{2}{}_{pred}$)	0.9057
Coefficient of variation (CV)	7.06

## Data Availability

The data presented in this study are available upon request to the first author.
